# Virtual diversity: Resolving the tension between the wider culture and the institution of science

**DOI:** 10.1177/03063127241263609

**Published:** 2024-07-29

**Authors:** Harry Collins, Robert Evans, Luis Reyes-Galindo

**Affiliations:** 1 School of Social Sciences, Cardiff University, Cardiff, UK; 2 Department of Social Sciences, Wageningen University, Netherlands

**Keywords:** expertise, interactional expertise, virtual diversity, standpoint epistemology, democratization of science

## Abstract

There are widespread calls for increased demographic diversity in science, often linked to the epistemic claim that including more perspectives will improve the quality of the knowledge produced. By distinguishing between demographic and epistemic diversity, we show that this is only true some of the time. There are cases where increasing demographic diversity will not bring about the necessary epistemic diversity and cases where failing to *exclude* some voices reduces the quality of the scientific debate. We seek to resolve these tensions with an analysis that turns on the way the experience-based expertise of non-scientists can be absorbed into mainstream science. Mostly it has to be done via what we call ‘virtual diversity’, in which scientists take responsibility for acquiring interactional expertise in the non-scientific expertise-based domains which they consider provide knowledge valuable to the science. We argue that virtual diversity represents the only feasible option in most scenarios, with cases where demographic diversity or full cultural mergers provide the solution being the exception rather than the rule. This analysis is an exercise in the sociology of knowledge, which is considered as being continuous with philosophy. The paper is prescriptive as well as descriptive, and the moral, cultural, political, and educational implications of the argument are drawn out. A main conclusion is that the acquisition of virtual diversity should be a new norm for science, allowing the voices of experienced non-scientist citizens to be heard but without eroding the institution of science, which continues to be a vital foundation of truth in democracy.

## Introduction

Calls to increase the demographic diversity of science often conflate two distinct claims. One is that increasing the demographic diversity of science in order to better represent the wider society is a matter of social justice. This is unarguable and efforts to achieve this should be supported.^1^ The second claim is that the inclusion of more perspectives that such demographic diversity creates will improve the quality of the knowledge produced. This claim is more complex: While it is true occasionally, it is also the case that failing to *exclude* voices from the scientific debate can lead to the opposite effect, preventing closure and harming citizens who might otherwise have acted differently. Here we seek to resolve this tension, developing the idea of ‘virtual diversity’ to explain how new knowledge belonging to non-scientists can be included within scientific debate but without overwhelming it in a cacophony of misinformation and filibustering.

We start from the assumption that science is an elite institution in the sense that entry depends on the demonstrable acquisition of specialist expertise. At the frontiers of research, these expertises are so esoteric that only a small number of people can acquire and practice them in ways that will be trusted by fellow specialists. Even after the frontier has sedimented into ‘the normal’, scientific specialties remain narrow. On the other hand, science aspires to be ‘universalistic’, open to participants from any cultural background, and giving rise to findings that are valid, if not necessarily comprehensible, across all national cultures. Work in science and technology studies (STS) has shown that these aspirations cannot always be fulfilled, so to save potential misunderstanding let us state now that: (a) an institution is not defined by what it invariably achieves but by its aspirations, and (b) universal validity does not imply that cultures and natural environments are the same everywhere, nor that technological policies should be the same everywhere; universal validity means that scientists should (aspire to) agree about where and why these things do and should differ.

The elite nature of science and its universalism have been questioned since at least the 1960s. It has been said that the community of science is insufficiently diverse to give rise to the desired universalism, either ignoring the expertise of those who are not traditionally members of the elite or paying no heed to the rights of unqualified stakeholders. These concerns were originally raised via organizations campaigning for socially responsible science, with similar themes appearing throughout the STS literature.

Crucially, responses to this can be framed either as attempts to address the social injustice of demographic exclusion or the epistemic injustice of discounted expertise, or both where it is a social injustice that gives rise to the epistemic exclusion.^2^ Responses to these claims typically argue that groups who are currently outside the scientific elite should participate more fully in the scientific research process, resolving the epistemic weakness if there is one, or, at least, ameliorating the social injustice. Outside groups who it has been said should be brought inside science include skilled workers with specialist skills (Wynne, 1992), sufferers from certain medical ailments who have expertise relevant to the related science as a result of their experience (Arksey, 1998; Epstein, 1996), indigenous groups who have relevant specialist local knowledge and local interests (Fibieger Byskov, 2017; Ludwig & Poliseli, 2018) and likewise local groups in Western societies who have special knowledge of conditions near industrial plants and the like (Irwin et al., 1996; Ottinger, 2010), women and other disadvantaged groups, who are underrepresented in most scientific research yet have specialist contributions to make (Subramaniam et al., 2016), and members of society in general, which would make science more democratic and could also make sure that scientific research is done in accord with general societal values (Douglas, 2009). What links these different examples is their emphasis on ‘democratizing’ science by making its boundaries more porous. This creates an opposed concern that, if unchecked, the boundaries will become so permeable that the institution of science will be eroded away (Collins & Evans, 2002).

The challenge is to provide a rationale for deciding how and when including certain outside groups might improve the quality of science and when it might make things worse. This has been referred to as the problem of extension (Collins & Evans, 2002) but we note below that a similar point is made by feminist philosophers of science such as Harding (1995a) and Longino (2002). The problem has become more pressing with recent developments in politics which are consistent in many ways with the ‘democratizing’ arguments of STS, such as ‘Trumpism’s attempts to erode the idea of truth and undermine the role of scientific experts in Western society. We have argued elsewhere (for instance, Collins, 2023; Collins & Evans, under submission) that a universalistic science is a crucial institution in resisting this trend and defending the idea of the truth.

Here, however, we want to explore the relationship between increasing diversity and the preservation of science as an institution. It is widely accepted that too little diversity can limit the capacity of science to adequately test its own assumptions but the opposite claim—that too little control over the borders of science risks losing the aspiration to universality—is more controversial, at least in STS. For example, does support for indigenous knowledge include the hope that it will, one day, become part of mainstream science or does recognition of its value imply that different modes of science should remain in perpetuity. The second choice would mean that science as we currently know it would be replaced by a collection of local sciences, each tailored to the interests of its supporters, something that would fit all too readily with populist politics.

We think diversity within science can be increased without sacrificing the aspirations that make science a distinct and vital institution. Where epistemic problems are related to a lack of diversity, but a full merging of inside and outside cultures is impossible or undesirable, they may be resolved, given the right conditions, through virtual diversity in which scientists take on the role of the other via the acquisition of interactional expertise. Virtual diversity is not easy and has to be pursued with the same assiduousness as the search for correspondence truth about the physical world. On the other hand, it has the potential to solve epistemic problems in ways that simply promoting demographic diversity cannot. We therefore propose that the aspiration to achieve virtual diversity should be established as a new constitutive norm of science.

In what follows, we will show how these problems work out in practice via case-by-case analysis, illustrating our points by revisiting a number of, mostly, well-known cases and providing something like a checklist of questions and answers for each. We think the right starting point for analysis of these complex problems is an understanding of expertise, especially the notion of interactional expertise. The next two sections of the paper summarise, first, the approach of Studies of Expertise and Experience (SEE) and, second, the notions of interactional expertise and virtual diversity. There then follow sketches of case studies under seven numbered headings, each followed by checklists that explore the extent to which social injustice is associated with epistemic damage. Prior to the conclusion there is a more extended treatment of the problem of extension.

## Studies of Expertise and Experience (SEE)

**Figure 1.**
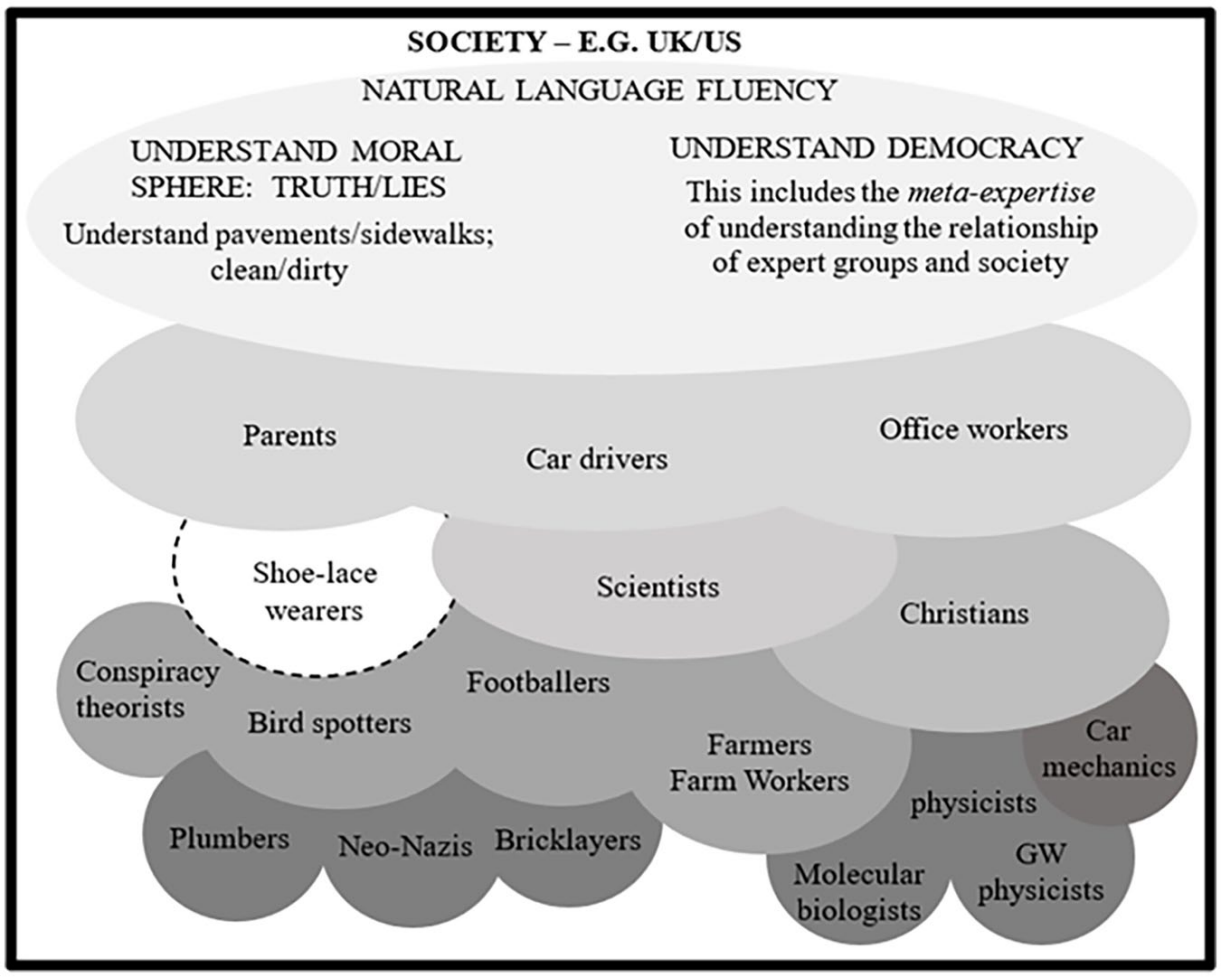
The fractal model of society (adapted from Collins et al., 2022).

The approach to the problem taken here depends on the analysis of expertise. The ideas have been under development since the 1970s, but from around the turn of the century they have come under the heading of ‘Studies of Expertise and Experience’ (SEE). The ideas now include the following:

a) Expertise and culture are coextensive. Both are acquired through socialization. There is ubiquitous expertise, such as natural language speaking and moral understanding, which is found in entire societies and is mostly acquired in early socialization, and there is specialist expertise, exemplified by the sub-specialisms of the natural sciences and other activities, which is acquired through tertiary socialization via apprenticeships or similar extended participation in the relevant group.
b) A society can be represented by the fractal metaphor: A society is a series of complexly, multi-dimensionally, and mutually embedded, culture/expertise-based groups from the top level of ubiquitous expertise through a series of more and more specialised activities, down to the bottom level of narrow technical specialisms (as in Figure 1). An individual is constructed out of periods of socialization in a set of such groups and individuals differ according to their experience, though all members of a society share the experiences that lead to that society’s ubiquitous expertises.
c) Each such group is associated with its own specialist spoken language. The social ‘groupness’ of any set of persons can, in principle, be tested in an ‘imitation game’ which asks a non-member to pass as a member in a verbal comparison judged by a fluent and demanding interrogator (when referring to the imitation game in this general sense, we use lower case; when referring to the Imitation Game as a method of social research, we capitalize it). If passing is hard and failure is possible, then this is a group with its own language not just a set of individuals. Gravitational waveform calculators and parents are groups; shoelace wearers and brown-haired people are not. Group languages are usually associated with a range of practices. Sometimes these will be narrow technical practices, sometimes just the ubiquitous, but often tacit, social understandings arising out of widespread practices that make properly designed Turing Tests so hard for computers to pass. These languages are not always inter-translatable, but they can be understood through extended immersion in the spoken discourse. It is possible to understand more than one mutually untranslatable language. Fluency in such a language is called ‘interactional expertise’. Acquisition of interactional expertise does not confer the ability to carry out the associated practices, though it does confer enough understanding of the practices to make sound judgements and to coordinate diverse practices in a division of labour. Ability to practice is known as ‘contributory expertise’, though the boundary between interactional and contributory expertise can be fuzzy, for example when the medium of the practice is, itself, linguistic.
d) One reason for the fuzziness of the boundary between interactional and contributory expertise is that the ability to converse fluently in a domain’s technical language—to have interactional expertise in respect of the domain—is to have experience of something that others do not have; it is coextensive with understanding the practical experiences pertaining to the domain even though it does not confer the ability to directly execute the practices. It does, however, enable its possessor to do things, such as make technical judgements, that others without the same level of understanding cannot. That is why technical managers, who do possess interactional expertise but cannot carry out any of the practical procedures pertaining to a domain, can nevertheless *contribute* to it (the same applies, for instance, to sports coaches). These distinctions are important if the relationship between human biology and culture are to be understood.
e) The difficulty of inter-translatability creates problems of many kinds. The problem of interdisciplinarity in the sciences is one of them. This is often referred to in terms of ‘trading zones’ with the original discussion being that of Galison (e.g. 1997), who considered the way chemistry and biology combined their languages and practices to form the newly merged discipline of biochemistry. One way of resolving these problems without a full cultural merger of the disciplines is the ‘ambassadorial model’: one or two ambassadors immerse themselves in the discourse of the other group and acquire interactional expertise. They are then able to represent, if not explain, the thinking of the other group to those in their own group (explanation requires translation). Though the ambassadors may not be able explain or execute practices they may be able to coach others’ learning of alien practices and languages.^3^

## Interactional expertise and virtual diversity

We are going to make the argument that the diversity needed within the scientific community can be, often is, and usually has to be, achieved through virtual diversity based on the acquisition of the relevant interactional expertise by members of the existing scientific community. Where many domain-specific perspectives and standpoints need to be included in a scientific domain, direct representation of each one by scientifically socialised members of each group is likely to be impossible. As these cases are the majority, virtual diversity will be the only way forward in most scenarios. This suggestion will be more or less resisted depending on the extent to which critics believe that understanding is irretrievably tied to embodied experience, or even biological constitution, and not language. The approach of SEE, which stresses interactional expertise, is at the other end of the spectrum.

Here we find the sociology of scientific knowledge contributing to the much wider argument about which groups can understand and represent which other groups in their analyses and writings. A wide-ranging discussion by Walker (2022) introduces the term ‘body politics’ to represent the position that the experiences of particular groups can only be understood and represented by those who share the body-type that gives the group its identity. As an example, Walker includes the attack on artist Dana Schutz for her sympathetic painting of the open-casket funeral of victim of lynching, Emmett Till. He puts forward a case very much aligned with what we are arguing here, although his analysis is more concerned with literature and the arts, where imaginative empathy has a greater role than it does in social science.

We are not alone in this rejection of identity politics as a rationale for insisting that only direct representation will do. Even amongst philosophers of science who argue for the importance standpoint epistemology, there is a recognition that contributions to an expanded analysis should not be limited to those who share body types or social groups. For example, Harding reflects on her own work as follows:

[s]tandpoint theory is not calling for phenomenologies of women’s worlds, or for ethnocentric (gynocentric) accounts. Nor is it arguing that only women can generate feminist knowledge; it is not an ‘identity politics’ project. Men, too, can learn to start their thought from women’s lives, as many have done. (Harding, 1995b, p. 343)

Here Harding is accepting the possibility of what we are calling virtual diversity.

Virtual diversity may be a new term but it describes a traditional and very familiar methodology and widespread type of social relationship. Much like trust, virtual diversity is both invisible and everywhere. Even in professional social science, nearly all the emphasis is on the difficulties of taking the view of the other rather it being an essential and central feature of human societies. Taking the view of the other is fundamental to the social sciences, such as anthropology and ethnography, but its vital role in societies is made more obvious when thought of in the context of the fractal model. Virtual diversity is what enables the various cultural domains of society to cohere. Of course, failures of virtual diversity and deficiencies of interactional expertise, especially when they are not recognised, cause many well-known and heavily discussed problems. It is the emphasis on these that obscures the larger point, that huge amounts of routine social interaction, and the very existence of large societies, depend on shared understanding.

In terms of the fractal model, we can start by explaining virtual diversity at a small scale—the division of specialist labour—and move upwards. Any complex division of labour depends on the coordination, and therefore mutual understanding, of the work of others when the persons who have to understand others’ work cannot do the work or, at least, have not done the work. The way that language and practice interact in the division of labour is illustrated elsewhere – see Collins, 2011, Figure 2. That figure shows why interactional expertise is so important and powerful: It must convey a practical understanding of others’ activities to allow co-ordination among them. Therefore, in that sense, the language *contains* the practice. It also shows why an outsider with no practical skills can come to understand the practices—because that is what each of the practical experts from within the domain have to accomplish in respect of nearly all their colleagues’ practical expertises.

Since cultures at every level nearly always involve distinctive practices as well as specialist languages, and the specialist languages will make continual reference to the practices, it is convenient to refer to these sub-discourses as ‘practice languages’. Moving up the fractal model from detailed technical division of labour, less specialist shared languages are the way higher level, less specialist groups, represented by the mid-level or even higher ovals in Figure 1, coordinate their contributions to society as a whole.

Virtual diversity, as already indicated, is also the stuff of the social sciences. Without virtual diversity, every criminologist would have to be a criminal and there would be no such things as sociology, ethnography, and anthropology where members of one social group reach into another in order to understand it. To see the problem at the most general level, Newtonian physicists wanting to understand relativity have the same problem as anthropologists wanting to understand an isolated Amazon tribe; toxicologists wanting to understand whether farmworkers are in danger when spraying powerful herbicides have the same problem as male doctors working as gynaecologists; social policy specialists trying to understand pensioner poverty have the same problem as architects trying to design prisons; and novelists trying to understand computer game addicts have the same problem as childless people trying to understand the parents of newborns. There are as many such problems as there are ways of combining any two ovals in the fractal model.

Moving to the arts (which is also the main topic of Walker’s, 2022 piece), without virtual diversity there would be no works of art in our lives that represent the life worlds of groups to which the artist does not belong and we would inhabit only the isolated world of our own experiences and imaginations; every ambitious artist or writer would suffer the same ignominy as Dana Schutz and for sound reasons. Without virtual diversity, science as an institution would cease to exist because the central tenet of universality—that no scientific claim is ruled out because of the ascribed characteristics of the scientist—requires the ability to share perspectives not body types. We now know, of course, that the idea of universality has to be thought about much more carefully given the idea of experience-based expertise but the claim that only certain kinds of people can do certain kinds of science would be disastrous should it become the default position: Instead of science we would have a series of sciences belonging to different social groups; standpoints would be petrified rather than means to greater shared objectivity. Furthermore, in the wider society, there would be no justice, but only ‘justices’, each science and each justice serving its own isolated community of understanding.

Without interactional expertise, interdisciplinary work within science would be impossible without a full-scale merging of the disciplinary cultures; distinct sciences would have to merge or, in the case of outsider groups, they would have to become scientific specialists in the domain in question as well as bringing their own standpoints to bear.^4^ It is only in rare cases that the scientifically unqualified can acquire sufficient technical and cultural understanding of science to join the community and, as already intimated, it is hard to develop the trust required in scientific work if one of the cultural parties has not had a scientific socialization. These things are not impossible, but they are hard, and it would be wrong to make them still harder by insisting on a full merger of cultures every time. Interactional expertise and virtual diversity provide a better solution to the problems of interdisciplinary and the inclusion of experience-based expertise.

### The reach of interactional expertise

The sociology of scientific knowledge has important consequences for knowledge as a whole. As a result of studies of science, we have discovered how central language is to scientific culture and to culture as a whole. The approach taken here is that, in principle—though not always in practice—fluency in any human practice language is attainable by any other human through long-enough and extensive enough immersion in, and interaction with, the spoken discourse. In addition to personal ability, enthusiasm and energy, the conditions for acquiring interactional expertise in other cultures include: (a) the group must have a rich language pertaining to the aspect of expertise that is to be acquired, (b) the hosts must be willing to allow the acquirer to immerse themselves in the discourse—to join in their social lives, and (c) the acquirer must have the resources to maintain presence in that group for an extended period and must be able to return unscathed.

The default assumption is that among human language-speakers, biological characteristics are only coextensive or near-coextensive with social boundaries as a matter of historical contingency—brown skin, ‘yes’, brown hair, ’no’. The Imitation Game test of groupness is linguistic, however, so it does not imply that biology cannot have an influence on *contributory* expertise (e.g., only biological women can give birth).

This is a strong claim: it says that, *in principle*, among humans, so long as a practice language exists, there are no experiences or cultural capabilities the understanding of which is ruled out by biological differences between human groups or by the differences in experience between different groups. If humans are capable of talking about their experiences, as they nearly always are, all experience-based expertises are permeable to anyone given enough time, the necessary resources, and good will on all sides. There may be cultural obstacles supported by biological differences but given enough determination on both sides of the divide, these can be overcome in principle; in terms of mutual understanding, biology is always subservient to culture. Thus, women can understand men and men can understand women if both sides want it to be the case and put in enough effort and resources, and Blacks can understand Whites and Whites can understand Blacks if both sides want it to be the case and put in enough effort and resources. Likewise, scientists could understand the perspective of experience-based experts if both sides want it to be the case and put in enough effort and resources; crucially, however, in this case there is no expectation that the non-scientists become fluent in the language of science. Properly designed Imitation Games with appropriately experienced populations ought to reveal these possibilities, which would support the contemporary view of the permeability of the boundary between men and women. To repeat, however, this does not mean that biological males can give birth to babies: That, in so far as it requires expertise, is clearly a matter of contributory not interactional expertise.

These arguments do not apply once we move beyond the realm of the human because the key to virtual diversity is human-like language; animals cannot possess interactional expertise. Those social scientists and philosophers who emphasize the continuity between humans and animals should recognise that the basis of human societies is the extraordinary depth and richness of human language and that animal means of communication do not provide the same possibilities for wide coordination of disparate actions and understandings across a range of specialist activities. Animals and other non-human entities do not possess human-like language and do not possess human-like culture.

### Wider significance of virtual diversity

An attempt is made to prove, or, at least, significantly increase the credibility of, this position—that humans from one group can in principle understand humans from any other group—in Collins (2020). One case examined in that paper is that of Rachel Dolezal, the White woman who claimed to be Black and who, in 2014-15, came to be chapter president for the *National Association for the Advancement of Colored People* (NAACP) in Spokane Washington. A similar point can be made using Reni Eddo-Lodge’s *Why I’m No Longer Talking to White People about Race* (Eddo-Lodge, 2020), which was raised by a critical referee of the first draft of this paper. Eddo-Lodge describes the incomprehension she experiences when talking to White people about the prejudice Black persons encounter in their lives. And yet Eddo-Lodge herself accepts the possibility of virtual diversity. She says: ‘I’m no longer engaging with white people on the topic of race. *Not all white people*, just the vast majority who refuse to accept the existence of structural racism and its symptoms’ (Eddo-Lodge, 2017, emphasis added).

Later in the same, *Guardian*, piece she discusses the murder of Black teenager Stephen Lawrence and its flawed investigation by the police, but she commends the (White) Sir William Macpherson, the high court judge who chaired the subsequent judicial inquiry and found that the investigation was ‘was marred by a combination of professional incompetence, institutional racism and a failure of leadership by senior officers’. This institutional racism, the report explained, is:

the collective failure of an organisation to provide an appropriate and professional service to people because of their colour, culture, or ethnic origin. It can be seen or detected in processes, attitudes and behaviour which amount to discrimination through unwitting prejudice, ignorance, thoughtlessness and racist stereotyping which disadvantage minority ethnic people. (Macpherson [1999], quoted in Eddo-Lodge, 2017)

Macpherson is here exemplifying virtual diversity.

Our work, to repeat, stresses the ability to acquire expertises/cultures of all humans, and therefore the potential cultural equality of all groups of humans. It recognizes, however, that biological difference is often deployed, consciously or unconsciously, to destroy the conditions that would lead to mutual understanding among the majority of group members. This is not a problem that we are trying to solve here, but we are proud of countering any claim that holds that there is some deep epistemic problem that keeps groups of people separate. Sir William Macpherson was, of course a member of an elite group of experts in the law, driven by the aspiration that justice should be blind—a member of an elite group like the scientists who we are discussing.

The same epistemic analysis bears on questions of representation and understanding that are central to many professional advocacy roles, including legal representation, social work, mental health and many other welfare-related settings.

### Dangers of virtual diversity

The most obvious peril of the idea of virtual diversity is that it will be done badly but still used as a warrant for exploitation. The concept invites a ‘virtual diversity lite’ interpretation (as discussed in Collins & Evans, 2015), in which some superficial conversation with unfamiliar groups or groups with unfamiliar expertises makes the investigator feel they have acquired enough interactional expertise to speak for them authoritatively. Furthermore, the idea, if handled insensitively, especially in the case of indigenous cultures, will bring out fears about colonialism and domination: ‘Here is the outsider coming into our society and purporting to understand it sufficiently well to recommend how we should live our lives.’ This is an old problem and one that cannot be dodged. After all, recommending MMR vaccination, or even Covid vaccination, is a kind of imperialism: It is a matter of an elite telling ordinary people how to live their lives. But if we want scientific medicine and the other benefits of science, political as well as substantive, we have to accept the tension and find ways of dealing with the complexities it creates. Case 5 in the next section illustrates how this might be accomplished. There is no Pareto solution if a simple model of democracy and liberal rights are valued on a par with the life and health of children; it is a choice the state has to make.

The moral dilemma is solved under the SEE banner by giving a special responsibility to an institution that has the formative intention to discover the truth and has learned that to discover truth as a community one must tell the truth (see e.g. Collins & Evans, 2017; Collins et al., 2020). Virtual diversity involves discovering the cultural truth about initially alien groups, and it has to be accomplished with the same assiduousness as the pursuit of truth in the rest of science. The acquisition of virtual diversity is proposed as a new norm of science, one that aligns with science’s formative aspiration of discovering the truth. It has to be treated with as much reverence and perseverance as the other norms of science even though the ‘correspondence truth’ involved—understanding and telling the truth about other cultures—is a matter of understanding the social, not the natural, world.

## Illustrative case studies

We now explain the idea of virtual diversity through its use in the analysis of the relationship between elite science and potential outside contributors. Drawing on a series of examples, each analysed in terms of the role of the expertise that is contributed by the cultures that come into contact, we examine whether, and to what extent, scientists were able to acquire expertise in other domains. Anticipating, we will find that full mergers are difficult and rare unless both cultures have already been scientifically socialized, in which instance it is essentially a matter of interdisciplinarity. We will find that, occasionally, scientifically unsocialized persons can acquire scientific expertise but usually the onus is on scientists to acquire interactional expertise in respect of experience-based experts. We will find that ameliorating social injustices often has no effect or negative epistemic consequences and is only sure to have positive epistemic impact under special circumstances. We will conclude that such special cases should not be treated as paradigmatic for all science. We will find too much stress on ameliorating actual or perceived social injustice at the expense of prior epistemic precaution can harm citizens. We will find virtual diversity is the only way to acquire understanding if the relevant experience is rare, as it usually is. We will find political activism can lead to positive epistemic effects if backed up by scientific understanding but to negative epistemic effects and subsequent harm where political power ignores or misrepresents scientific expertise. We are illustrating this with a series of, mostly, well-known case studies because the well-known studies have acquired an iconic status in STS. What we want to do is cause the community to reconsider whether the studies that they think of as standing for so much really do represent so much. We will start with the Rosalind Franklin example precisely because it doesn’t represent much if anything in terms of epistemic consequence even if it does mean a lot in terms of social justice. That is the distinction we are trying to bring out.

### The statistics of scientific specialties and the demographic solution

Before looking at the case studies, however, let us explain in more detail why the demographic diversity solution does not work most of the time. This is simply a consequence of the number and range of scientific specialties. Consider the case of social injustice that, for better or worse, has gained almost mythical status: the case of the post-Chernobyl Cumbrian sheep farmers. According to Wynne (e.g., 1992), the farmers’ experience-based expertise was excluded from the scientific debate about the treatment of grazing sheep after the irradiation of the Cumbrian fells, because of the farmers’ low social and scientific status. But let us imagine that British society was evened out in the fashion of a socialist utopia such that sheep farmers were now a high-status group appropriately represented according to their numbers in the population in a prestigious profession such as scientific research. They are still a small group of experts, not much larger than the group of radioactivity experts, and the odds of an ex-sheep farmer or a sheep-farmer’s offspring being among the group of scientists involved in the particular analysis of the post-Chernobyl Cumbrian fells are vanishingly small. On the other hand, even if the sheep farmers had had higher status and been welcomed into the discussion, their lack of scientific socialization and, therefore, their inability to converse with the scientists, would still have to be solved via interactional expertise. Under our proposal, the responsibility for this would lie with the scientists, one or more of whom would have to enter the sheep farmers’ world and, like ambassadors, bring their tacit understandings of sheep ecology back into the science that was informing policy-making.

The same applies to rare medical conditions, particularly where they occur predominantly in old age, or where they have high fatality rates. In these cases, the chance of being treated by a doctor who has experience-based expertise in the particular ailment is almost zero, whatever the demographics of the medical profession. Nearly all the experience you are going to get applied to your medical treatment is going to come via interactional expertise: Doctors get nearly all their experience concerning the conditions you are likely to suffer from by talking about them—perhaps with other doctors, often with expert patients who are not doctors—but rarely, if ever, from direct personal experience of the actual illness. In so far as doctors are experience-based experts, it is experience in medical settings that they possess, not the experience of expert patients.

The same usually applies even where the disadvantaged group in question is large. Consider women. Gynaecology will benefit in terms of the scientific knowledge that it gives rise to as demographic dominance in the profession shifts from men to women (as seems to be happening), because it means women undergoing gynaecological procedures are much more likely to be treated by specialists with first-hand experience of the problems. Nevertheless, for many gynaecological conditions the chances that even a female doctor will have experienced the particular patient’s ailment remains small. Where those ailments are rare, such as molar pregnancy or bicornuate uterus, they are vanishingly small. This is not to say that a medical profession that reflects either the wider society or its particular patient group more closely is not a worthwhile ambition. The point is simply that solutions to social injustice won’t necessarily resolve scientific knowledge problems, even if they make the environment more hospitable to specialists from the disadvantaged groups.

To express the point of the case studies in a way that a critical referee would have preferred us to express it:

Not every case of exclusion results in epistemic injury and not every case of inclusion results in scientific or social benefit. Cases in which outsiders can meaningfully acquire interactional expertise and really become part of the process, while celebrated in the STS literature, are probably rare. Therefore, it’s incumbent on the scientists to do the interactional work themselves and then determine what to include or exclude.

#### Rosalind Franklin

Rosalind Franklin’s work using X-ray crystallography was foundational to the discovery of the double helix, but by their own account Crick and Watson stole it and used it to their own advantage. Many believe that Franklin should have shared the credit, and, if she had lived, the Nobel prize, for the discovery. A recent interpretation suggests that the standard story promulgated by Crick and Watson is untrue and that Franklin cooperated fully with them, but even if that is the case, the standard account works as a thought experiment and we will proceed as though it was the case.^5^

Franklin, on the standard account, suffered a social injustice that is indicative of the injustices long inflicted on women in science. But our question is whether this injustice undermined the quality or value of the *science* produced in the discovery of the double helix. The answer seems to be ‘no’. While Crick and Watson’s willingness to proceed without acknowledging Franklin’s contribution may have mirrored the widespread prejudice and discrimination against women in science, the quality of Franklin’s scientific work was clearly recognised by Crick and Watson—that is the very reason they used her results (whether stolen or not) and, as far as we know, no-one has since claimed that Crick and Watson’s claim about the double helix was flawed because a woman did or did not contribute to it.^6^ Something similar might be said of almost the entire history of physics.

**Table:** 

Groups involved	Molecular biology and crystallography within science, men and women from wider society
Injustice and knowledge	Social injustice did no epistemic damage
Politics	No obvious effect from or on wider society at the time
Who was affected	Established scientists
Crucial actors	A few established scientists
Conclusion	Social injustice is not necessarily epistemically damaging

#### Early primatology and reproductive science

In spite of the statistics that affect most specialties, there are sciences in which the demographic cultural position of the group as a whole does have an epistemic bearing. Primatology and reproductive science are an exception to the rule that one is unlikely to encounter a specialist in the ‘condition’ being investigated by adjusting the entry conditions to the profession. This is because in these cases, the ‘specialist’ expertise is simultaneously a ubiquitous expertise. That means, for example, that the social injustice experienced by women within science and in the wider society can have a direct effect on the science.

For example, early primatology studies were conducted by men and tended to focus on stereotypical male behaviours such as aggression in species where males tend to be the dominant sex.^7^ The behaviour of female primates in these settings, or of species in which females played a bigger role (i.e. matriarchal not patriarchal groups), were simply not studied, and behaviours observed in the patriarchal species were generalized as typical, natural and normal traits. As female researchers began to enter the field, they challenged and changed its research practices by studying different species and by paying more attention to the role of the females within the group. The result was that a much wider range of social structures were identified within primate species and a much wider range of behaviours documented. This, in turn, undermined the claims made by the male-dominated researchers that the behaviours they had recorded were natural and normal, an argument that was often put in an evolutionary context to justify and normalize similar traits in humans.

A similar addition of complexity and nuance occurred as female researchers began to enter the domain of reproductive science. Here the influence of male dominance was reflected in the gendered language used to describe the different characteristics of the egg and sperm (the classic account is Martin, 1991). Thus, the female egg was seen as being essentially passive and doing little more than waiting for the sperm. In contrast, the sperm was highly active and agentic, both in the way it was seen to compete to reach the egg and also in the way that it was seen as penetrating the membrane in order to fertilize the egg. Over time, perhaps as female researchers entered the discipline or perhaps due to increased awareness of gendered language, the characteristics assigned to egg and sperm began to change, with the egg being seen as a more complex entity that played a more active role in selecting and enabling the sperm to fulfil its function. Nevertheless, gendered language continues to appear in medical and biology textbooks (Campo-Engelstein & Johnson, 2014), demonstrating how difficult it can be to change deep-seated cultural assumptions.

We have treated these two cases together because they illustrate the same point, which, to repeat, stands in contrast to the majority of the examples discussed and the majority of cases which could be found in science. The reason is that in these cases the specialist expertise that had been lacking and led to negative epistemic consequences was, at the same time, a ubiquitous expertise among the excluded group, and that means that including that group had a good chance of remedying the problem. Just including the view of the world from a female perspective, as that perspective is most generally found, or at least represented, in Western cultures, rectifies the narrow view caused by the pre-existing social injustice.

It may be that there are other cases that are not dissimilar from the one of women. Where the proportion of Black citizens in a country is large, it may be that more Black scientists would make a difference to Black epistemic concerns. Going back to Eddo-Lodge, among the Macpherson report’s recommendations was that the police force boost its Black representation, and that all officers be trained in racism awareness and cultural diversity. Though Afro-Caribbeans represent only 3% of the population of the UK, one can still make sense of Macpherson’s recommendations, given that we are talking of ubiquitous expertise—moral judgements and the like—not specialist expertises.

But these are not paradigmatic cases of distorted science; they are the unusual ones. It is probably not a coincidence that these are also the sciences where much of the impetus for the early work in feminist and standpoint philosophies of science emerged. The paradigmatic cases are those where both experience and special expertise are both narrowly distributed. In such cases the likelihood of encountering a specialist who has anything other than virtual experience of the state of the world being investigated will be slim. To repeat, the very influence of changing the gender balance of those working in primatology and reproductive science is an exception that proves the rule because it is a rare case of the coincidence of ubiquitous and specialist expertise related to the actual topic of the research.

**Table:** 

Groups involved	Men and women within specific scientific disciplines, men and women from wider society
Injustice and knowledge	Injustice was causing poor science until gender balance rectified it
Politics	N/A except perhaps for internal politics of science
Who was affected	Primatologists/ reproductive scientists
Crucial actors	All scientists in these fields
Conclusion	Injustice and epistemic damage can be resolved by changing gender balance in the science, but only if the relevant specialist expertise is more accurately classed as a ubiquitous expertise. These cases are untypical, rather than exemplary, and should not dominate thinking about the epistemic consequences of social injustice.

#### San Francisco AIDS activists

The AIDS activists’ successful campaign to influence medical research has been documented by Epstein (1996). Though Epstein claims the crucial intervention in the design of clinical trials was a matter of political activism, the activists acquired enough understanding of the science to be congratulated by the scientists they were initially opposing. We believe it was this high level of enculturation that was crucial to their success. It may have been the salience of the political activism that caused scientists to look at the claims seriously but, without the activists acquiring enough technical and methodological expertise in the science to propose viable new protocols in place of the double-blind tests to which they were objecting, we do not think they would have succeeded in modifying established practice. This, then, according to our analysis, is a case of full cultural merger between scientists and unqualified outsiders, the latter acquiring scientific socialization.

In contrast with the case of Rosalind Franklin, the expertise of the treatment activists was not immediately recognized by the mainstream medical community. Nevertheless, over time and through a combination of learning and political activism, the treatment activists were able to build alliances with those members of the medical research community who were also concerned about the design and conduct of clinical trials and, eventually, to have their concerns recognised as legitimate. The design and conduct of clinical trials were changed as a result and members of the activist community began to play a more official role within the medical research establishment.

**Table:** 

Groups involved	Medical scientists, well-educated AIDS sufferers
Injustice and knowledge	Injustice could have had a negative outcome but didn’t
Politics	Initial injustice was rectified by political activism but full cultural merger also was established by activists.
Who was affected	Expert patients
Crucial actors	The expert patients
Conclusion	Political action can be influential creating the conditions needed to recognize an injustice but a full cultural merger between initially scientifically unsocialized citizens and scientists is rarely achievable.

#### Ethnobiology

In the artisanal fishing village of Siribinha in Northeastern Brazil a multidisciplinary academic team carried out extensive enculturation and social science fieldwork looking at fishers’ expertise and the existing overlaps with scientific knowledge (see Renck et al., 2022). The researchers identified similarities and differences between fishers’ classification of species and scientific taxonomies. They revealed, for example, that local knowledge about fish spawning periods meant that seasonal policy restrictions on fishing were being implemented at the wrong time for that location. Changing this affected fishers’ lives positively and improved conservation efforts. Similar results have also been reported in other species and locations.

In terms of SEE, the ambassadorial work of shedding light on the traditionally neglected knowledge of fishers created the possibility of improving science through virtual diversity, bringing tangible benefits to the fishers but not requiring them to become scientists. Carrying out such work is clearly complicated and fraught with ethical complexity. The ontologies and epistemologies of indigenous and mainstream science come with wider sets of values that may be difficult to reconcile, thus giving rise to concerns about exploitation and extraction in which indigenous knowledge is simply mined for whatever the dominant science can use. Nevertheless, this case shows that, given sufficient care and reflexive awareness on the part of the scientists involved, the norm of virtual diversity can bear fruit.

**Table:** 

Groups involved	Established scientists (natural, social, ethno and philosophy), local experience based experts
Injustice and knowledge	Injustices rectified through virtual expertise with positive effects
Politics	Concerns about sustainable development put focus on indigenous communities and their role in managing and preserving ecosystems
Who was affected	Scientists, local fishers
Crucial actors	Ethnobiologists and philosophers acting as intermediaries and ambassadors creating virtual diversity
Conclusion	Virtual diversity can be created through careful and proactive efforts that avoid ‘extractive’ models of knowledge acquisition.

#### Cumbrian sheep farmers revisited

It seems clear that the Cumbrian sheep farmers in Wynne’s (e.g. 1989, 1992) study possessed experience-based expertise that would have made a valuable contribution to the scientific analysis as initially conducted by scientists from the UK Ministry of Agriculture, Fisheries and Food (MAFF). Unfortunately, it was ignored. Even if it had been recognized, as explained above, using that expertise would have required and still requires a crossing of cultures because sheep farmers are not socialized into the cultural aspirations of science and have no fluency in science’s specialist ‘practice language’. Even if the scientists, who were responsible for providing advice on how to handle the sheep on radioactive pastures, discovered that the sheep farmers had something to offer it would still need melding into their scientific culture and in this case full cultural merger would be an enormous undertaking. In the biochemistry case discussed by Galison (1997) and in the examples discussed under the second case above, it was made much easier because, at the outset, both groups shared the language and culture of science as a whole. The sheep farmers did not possess that fluency nor is it likely that they would have the time and inclination to acquire it by undergoing an apprenticeship in science. This is not least because of the ways in which they felt this mode of understanding threatened their own identities, which is the main emphasis of Wynne’s analysis.

Thus, if the cultural divide were to be crossed, it would depend on the MAFF scientists immersing themselves in the language of the sheep farmers and absorbing their understandings into the science. It would not require them to become sheep farmers, only to develop a level of interactional expertise.^8^ This level might be quite shallow, since the mistakes that the scientists were making in terms of sheep husbandry might not rest on much more than *information* about how sheep behave and the economics of sheep husbandry.^9^

**Table:** 

Groups involved	Established scientists, experience based experts (sheep farmers)
Injustice and knowledge	Injustice (in the form of class prejudice) did lead to negative scientific outcome, but it looks as though it could have been rectified easily with virtual diversity.
Politics	Generalized distrust of government scientists by local community as a result of past experience with nuclear industry that increased as post-Chernobyl restrictions changed without warning or explanation
Who was affected	Both groups, though sheep farmers suffered most as their livelihoods were directly affected by policy changes
Crucial actors	MAFF scientists failing to acquire virtual diversity; sheep farmers as relevant experience-based expertise
Conclusion	Checking for relevant experience-based expertise in order to create the virtual diversity needed to ensure epistemic standards should be the norm.

#### MMR

Where an existing consensus is challenged, this needs to be on the basis of evidence and expertise, and according to the norms and values of science. In the previous examples, these characteristics were either met by the activists themselves or by the ethnobiologists and philosophers acting as a bridge between the two communities. In the case of the measles, mumps and rubella (MMR) vaccine, however, these conditions are not met. Instead, we see a potential insider who fails to uphold the expected standards being supported by a range of outsiders who lack the kind of in-depth knowledge possessed by the treatment activists, sheep farmers and fishers, a clear example of the epistemic dangers that can arise from what, in our 2002 paper, we referred to as ‘the problem of extension’.

In the late 1990s, the medical doctor Andrew Wakefield broadcast the idea that MMR vaccine could cause autism. He was aided by *The Lancet*’s publication of his article (Wakefield et al., 1998), which was not retracted for another 12 years, and by the mass media’s preference for stories that mistakenly ‘balanced’ scientific evidence with parents’ claims about the cause of their children’s autism. The net result was a revolt against the MMR vaccine by a significant minority of parents that undermined herd immunity and led to repeated measles epidemics in many locations that continue to this day (e.g. Devlin, 2023). But though some, or even many, parents witnessed the agonizing onset of autism in their child shortly after an MMR vaccination, there was no evidence of a causal link even at the time. A reading of the original *Lancet* article reveals totally inadequate statistics and all the epidemiological evidence was against it. Later it would turn out that Wakefield had a financial interest in his claim and that his recruitment of cases was unethical. Sadly, a number of prominent figures from the STS field endorsed the parents’ view, which was very popular at the time in such circles, and still more sadly, as far as we know, now that the reality of the dangers of the revolt have revealed themselves in concrete form, none of the social science-based protagonists have ever reconsidered their one-time involvement and reflected upon their mistake; this damages the credibility of our discipline as a determined seeker after truth.

Unfortunately, the legitimation of a revolt against one vaccine is likely to be seen as supporting other vaccine revolts. This is why it is so important to disentangle sentiment for those outside the scientific elite—widely admired among social scientists—with actual epistemic damage. The epistemic damage in this case was a consequence of the championing of the views of actors marginal to the science but who were the most important stakeholders.

**Table:** 

Groups involved	Medical scientists, citizenry/social scientists/commentators, who all treated themselves as having the same epistemic rights as accredited scientists (problem of extension)
Injustice and knowledge	Imagined injustice led to negative epistemic outcome and greater injustice.
Who was affected	The defenceless, such as children and subsequent generations
Crucial actors	A small group of citizens (+social scientists, celebrities, journalists)
Conclusion	Imagined injustice can be dangerous as small groups can exert a disproportionate effect. This means elites, including the media, governments and scientists have important roles to play in communicating what is and is not known and holding each other to account.

#### Antiretroviral drugs in South Africa

A similar case of unhelpful and uninformed ‘participation’ in science, having a disproportionate and devastating effect, is the decision by South African President, Thabo Mbeki, supported by his parliament, *not* to distribute antiretroviral drugs to the population at the time of a growing AIDS epidemic (Weinel, 2007, 2010). Mbeki claimed that scientific research showed that the drugs were unsafe and inefficacious. Mbeki was acting as though his scientific understanding was superior to that of the scientific establishment. In fact, while a debate about the drugs’ efficacy and safety could be found on the internet, the scientists making the case against the drugs were no longer able to publish their views in regular journals. Mbeki’s scientific support, as with the MMR case, came from the scientific fringe, with opposition coming from both mainstream science and civil society groups.

In a backhanded way, the Mbeki case supports Epstein’s view of the importance of political power in the case of science. Mbeki and his colleagues were running the country and, unsurprisingly, they got their way, with, it is now understood, seriously damaging effects on, for example, babies born to mothers not given the drugs: Numerous babies were born HIV-positive, and the drugs could have reduced the incidence of this outcome. Here there was no full cultural merger between the established scientists and outsiders (including the fringe, some of whom had at an earlier time been members of the mainstream). Instead, there was a long process of campaigning and protest against the policy that involved mainstream scientists, civil society groups and journalists. Eventually, this led to the policy being reversed but not before many more children had been needlessly infected. There was no virtual diversity but none was needed: What we see here, as with the MMR case, is the problem of extension made real as knowledge claims that should be dismissed are taken seriously. Instead, what was needed was for Mbeki to make better social judgements about where the relevant expertise was located and to make better use of the expert advice that was available to him.

**Table:** 

Groups involved	Medical scientists, political leaders
Injustice and knowledge	Failure to correctly identify mainstream scientific consensus led to poor political decision-making and misleading justification of policy choice.
Who was affected	Pregnant women and their unborn children who were put at greater risk of developing HIV/AIDS
Crucial actors	Political leaders who failed to recognise relevant expertise; opposition from mainstream science and civil society that eventually forced policy to be changed.
Conclusion	Those outside the scientific community may struggle to recognize current consensus, with the result that too much weight is given to marginal or out-of-date ideas leading. Expertise is needed to recognize what current consensus is and what counts as reasonable questioning of this.

#### A wider perspective?

In the submitted version of this article we wondered if we might be accused of missing the point because more diversity in science at the outset would actually precipitate a different kind of science. For example, we asked whether we had been too narrow in discussing the consequences of the exclusion of Rosalind Franklin. One referee made the same point.

Maybe the recognition of her work would have allowed her to launch other research programs. Maybe she would have had considerable resources and make other discoveries. There is of course no way to know, and little sense in trying to do [so].

Given this we can only agree that attempting counter-factual speculation of this kind is just the problem that utilitarianism as an ethical philosophy cannot solve. In any case, we already agree that there should be diversity in science on moral grounds so there is no argument to be had on that issue. It is the demonstrable epistemic conclusions and justifications that we are discussing.

## The problem of extension as it applies to different kinds of cases

The most pressing problem addressed by this article is what can be referred to as, among other things, ‘the problem of extension’: Enthusiasm for bringing outsiders into science, while laudable for its democratic instincts and for its resolution of social injustices, risks destroying science as a distinct institution. This is because, as intimated, a current concern is the new fragility of science as a check and balance on the slide from populism to fascism, most graphically illustrated by the erosion of truth under potential dictators such as Trump. Once more, in this concern we echo philosophers such as Harding and Longino, both of whom champion the inclusion of wider groups into the scientific process, but in order to strengthen it, not destroy it. Harding advocates for the ‘stronger objectivity’ that can be created by the inclusion of more standpoints reflecting diverse opinions in the scientific debate, while Longino agrees that ‘the greater the number of different points of view the more likely it is that scientific practice will be objective … [and] … reliable’. (Longino, 1990, p. 80). Crucially, however, both recognize that expanding the number of opinions without limit is not good enough. Thus Harding writes:

Obviously not every starting point for thought that lies outside a dominant conceptual framework is likely to enlarge our understandings. We can agree with the defenders of weak objectivity that at least some of the interests and values they think should be excluded from directing knowledge projects do indeed retard the growth of knowledge- ‘Think of Nazi science!’ (Harding, 1995a, p. 25)

Harding does not pursue any methods for limiting the number of standpoints, however, but Longino (2002, pp. 129–133) goes further, attempting to solve the problem by defining four criteria that can be used to assess the ‘epistemic effectiveness’ of potential contributions and hence prevent what she calls the potential ‘cacophony’ of unchecked debate. Of these, the two which translate most directly into collective actions by the scientific community and wider society are:

*Venues*. There must be publicly recognized forums for the criticism of evidence, of methods, and of assumptions and reasoning.

*Public Standards*. There must be publicly recognized standards by reference to which theories, hypotheses and observational practices are evaluated.^10^

The approach to the problem of extension taken here shares these concerns but protects the elite nature of science by growing any expansion of science in respect of experience-based expertise from inside science, with scientists primarily responsible for taking into account the *epistemically valuable* expertises of the unqualified. Only when we turn away from epistemology to resource allocation, does the problem change to one which is more readily compatible with the fusion of ubiquitous societal choices.

Returning to what we have referred to as the problem of extension, this was, perhaps, first discussed in 1959 under Kuhn’s (1959, 1979) heading of ‘the essential tension’. The essential tension is between the consensus over assumptions that makes normal science productive and the introduction of radical novelty that allows for paradigm change; if it is all novelty and no acceptance of authority, science collapses. The approaches we are critically discussing here bring in new ideas from outside the existing established body of science: they are radical in that sense. As mentioned, we (Collins & Evans, 2002) introduced the related tension between the problem of legitimacy and the problem of extension when dealing with technological decision making in the public domain. This was to deal with questions about how to extend the right to contribute to elite science to the public in general. Calls to democratize science and improve its legitimacy by allowing the public to participate in its elite activities could not, we argued, make sense without also dealing with the problem of how far to extend these rights. Too limited an approach will fail to give science or the advice derived from it the necessary legitimacy but too liberal an extension and science as a distinctive institution disappears, leaving nothing but the politics of competing opinions. This dystopian outcome, in which the communities and social order represented in the fractal model collapse and are re-arranged in a new hierarchy fuelled by social media, is discussed in Collins et al. (2022).

The problem of extension, and hence the need to draw boundaries, is also clear in the case of fringe science. The consequences of mistaking fringe science for the real thing has already been illustrated in the case of the antiretrovirals, but there are other examples. Following Harding, for example, we would not want to include the antisemitic views that are sometimes used to bolster the rejection of relativity in the contemporary fringe of physics (Collins et al., 2017), as a standpoint which ‘strengthens objectivity’ but the same problem extends to the entire fringe whether racist or not. Thus, the preprint server arXiv, originally set up with the intention of being wide open, had to introduce ways of demarcating far-outlying views to prevent it being overwhelmed by alternative viewpoints (Collins et al., 2017). These are nowadays made available in the ‘alternative’ preprint server ‘viXra’.

Merely opening scientific discussion up to any group which has a view that does not fit with that of the powerful does not work as a solution to the problem of legitimacy or as a means to strengthen objectivity. It is certainly likely to generate views that do not coincide with those of the establishment, but without some criteria or process of quality control science would become a Tower of Babel. In sum, the endorsement of variety of standpoints—the more the better—only works as a philosophical idea so long as boundaries are set surrounding the legitimate possibilities. To call for the inclusion of more viewpoints without recognizing, as Harding and Longino do, that, *at the same time*, some solution has to be found to limit the envelope of possibilities, is philosophically incoherent and socially irresponsible. Instead, the rationale for the limit has to be presented at the same time as the plea for diversity; they are two sides of the same coin.

## Conclusion

We believe that SEE’s solution to the problem of how to limit the extension of scientific rights is a workable one: Give primary trust to claims about the observable world made by those who are institutionally and morally committed to the truth. Science is an institution the formative aspiration of which is to find correspondence truth and scientists have discovered that it is best achieved in small groups who trust each other because they know that the aim can be achieved only if they tell the truth.

We have brought out another feature of a trustworthy science, namely that when operating across cultures, scientists should put the same energy and assiduousness into achieving virtual diversity. Harding and Longino put the matter in terms of stronger objectivity arising out of multiple standpoints, we stress the need to bring in relevant experience-based expertises. The new contribution to the debate is the argument that this diversity should, in most cases, be achieved not by bringing outsiders into science but by bringing in their expertises via virtual diversity managed by the scientific community. No scientific community can ever be truly representative of all human groups, so, if we want to continue to aspire to maintain a universal science, we need to decide which kinds of diversity matter and how to include them.

Our proposal is also a partial solution to the most pressing political problem in the West since the Second World War and it grows out of the close examination of the social nature of scientific knowledge, starting with an understanding of specialist division of labour. Gender equality is always a social good, so we would want it for all science, but is only an epistemic good where gender is relevant (i.e. provides a specialist expertise) to what is being researched. The same principle would also apply to other topics and standpoints. Virtual diversity solves this problem by enabling specialist expertises to be included even when social justice or other conditions have not been met.

## Notes

1. This is not strictly correct: There are some who oppose these moves. One of the proximate triggers for this paper was the article by Abbot et al. (2023), who appear to want to re-start the science wars by creating an opposition between scientific merit and diversity. Though we share their belief that science should strive to be independent, we reject their conclusions, not least because they misrepresent the nature of science. Given the importance of the issues, it is crucial that we, and STS more broadly, provide a reasoned alternative that does not leave the field clear for a futile war between extreme and ill-informed views.

2. There are some overlaps here with the work of Fricker (2007) on epistemic injustice, with excluded groups at risk of both testimonial and hermeneutic forms of injustice. Apart from the focus on science rather than inequality, we differ from Fricker in that we are as concerned with identifying the circumstances in which discounting testimony is the right thing to do as we are with identifying when it is the wrong thing to do.

3. They may be able to provide guidance in terms of ‘second order measures of skill’ (Pinch et al., 1996). Galison (1997) provides the original discussion of trading zones, with Collins et al. (2007, 2017) analysing the problem in greater detail and introducing the ambassadorial model.

4. Already in the Third Wave paper (Collins & Evans, 2002), we suggested that this responsibility should fall on the scientists; here we are extending the argument.

5. Thanks to Jacob Stegenga for pointing out the more recent interpretation. Saey (2023) provides a popular account of it.

6. There are similar cases of stolen discoveries among men alone, such as is found in Krige (2001), but, if what we have referred to as the ‘standard account’ is correct, then the Franklin case had an additional gendered dimension.

7. This description is taken from the summary provided in Rosser (1992). Haraway’s (1989) *Primate Visions* provides a more complex analysis of primatology, locating its practices within a more nuanced and contested cultural field and examining how the stories told by primatologists—both male and female—contribute to contemporary discourses of gender, sexuality and family.

8. In his ‘Sheepfarming after Chernobyl’ (i.e. Wynne, 1989), Wynne does report some ‘serendipitous’ interactions between some scientists, who made repeated site visits to farms and even stayed on some as guests for several days at time. He reports (pp. 37–38) that the farmers were able to learn about the scientists’ world as a result of this, but says nothing about what, if anything, the scientists learnt about sheep farming.

9. Wynne does not report the results of any interviews with MAFF scientists. As far as we can see, he investigated only the levels of trust and respect for the scientists among the farmers. Unlike in, say, a courtroom, as far as we can see, only one side of the disagreement is represented. This is, perhaps, a symptom of starting the analysis from the point of view of (real or perceived) injustice rather than as a problem of the merging of cultures, which would always involve symmetrical effort in respect of both sides. We have approached Wynne (02/08/2023) in hopes of reviewing the case and any efforts to discuss matters with the MAFF scientists but he has not responded.

10. The other two criteria seem to be more about how the efforts to extend are received by the scientific community rather than prescriptions translatable into recommendations for scientific action.
